# Management challenges faced by administrators at dementia care nursing homes in China

**DOI:** 10.1093/geroni/igab046.3413

**Published:** 2021-12-17

**Authors:** Lin Jiang, Fei Sun, Robin Bonifas, David Hodge

**Affiliations:** 1 The University of Texas Rio Grande Valley, Edinburg, Texas, United States; 2 Michigan State University, East Lansing, Michigan, United States; 3 Indiana State University, Terre Haute, Indiana, United States; 4 Arizona State University, Phoenix, Arizona, United States

## Abstract

Currently, more than 10 million Chinese older adults have been diagnosed with dementia, a number that is expected to increase as the population in China rapidly ages. Yet, little research exists on dementia care in Chinese long-term care facilities. Our research addresses this gap in the knowledge-base by examining the challenges nursing home directors encounter as well as the coping strategies they employ to deal with these challenges. Twenty-one facility directors employed by faith-based nursing homes across 14 provinces in China participated in this study. Semi-structured interviews were conducted in respondents’ native language via phone or in person. Most respondents reported a religious affiliation, and about half were female and had been facility directors for more than four years. Two researchers fluent in Mandarin and English coded interview transcripts; thematic analysis was conducted to identify patterns in the data. The results indicated four primary challenges, which pertained to recruiting and retaining nursing staff, funding, lacking support from the government, and conflicts with family members. The coping strategies included obtaining and using external resources such as volunteer visitors, operationalizing personal spiritual beliefs, and providing training to improve skills and empathy among employees. This study contributes to nursing home practice by expanding our knowledge of culturally relevant dementia management strategies in China. Suggestions to address management challenges from a policy and practical perspective include clear and sustainable financial support from the government, staff training, and staff-resident ratio regulations, seeking external resources, and integrating spiritual strategies into problem management and service quality improvement.

